# *In Vivo* Imaging of Single-Molecule Translocation Through Nuclear Pore Complexes by Pair Correlation Functions

**DOI:** 10.1371/journal.pone.0010475

**Published:** 2010-05-03

**Authors:** Francesco Cardarelli, Enrico Gratton

**Affiliations:** Laboratory for Fluorescence Dynamics, Department of Biomedical Engineering, University of California Irvine, Irvine, California, United States of America; Clarkson University, United States of America

## Abstract

**Background:**

Nuclear pore complexes (NPCs) mediate bidirectional transport of proteins, RNAs, and ribonucleoproteins across the double-membrane nuclear envelope. Although there are many studies that look at the traffic in the nucleus and through the nuclear envelope we propose a method to detect the nucleocytoplasmic transport kinetics in an unperturbed cell, with no requirement for specific labeling of isolated molecules and, most important, in the presence of the cell milieu.

**Methodology:**

The pair correlation function method (pCF) measures the time a molecule takes to migrate from one location to another within the cell in the presence of many molecules of the same kind. The spatial and temporal correlation among two arbitrary points in the cell provides a local map of molecular transport, and also highlights the presence of barriers to diffusion with millisecond time resolution and spatial resolution limited by diffraction. We use the pair correlation method to monitor a model protein substrate undergoing transport through NPCs in living cells, a biological problem in which single particle tracking (SPT) has given results that cannot be confirmed by traditional single-point FCS measurements because of the lack of spatial resolution.

**Conclusions:**

We show that obstacles to molecular flow can be detected and that the pCF algorithm can recognize the heterogeneity of protein intra-compartment diffusion as well as the presence of barriers to transport across NE.

## Introduction

Macromolecular traffic between the nucleus and the cytoplasm is enabled by nuclear pore complexes (NPCs), large macromolecular assemblies that punctuate the nuclear envelope (NE): transport across the NPC not only localizes proteins destined to the nucleus or cytoplasm, but also plays a key role in signal-transduction pathways and in the regulation of major cellular processes (for review see [1], [2], [3]). The NPC is composed of ∼30 different polypeptides designated nucleoporins, which yield a total mass of ∼125 MDa in vertebrates [4], [5]. A large number of nucleoporins contain repetitive sequences, the phenylalanine-glycine (FG) repeats, which are intrinsically unfolded [6], and form a selectivity filter for the passage of molecules through the pore [7]. Molecules which do not specifically interact with the FG repeats, can cross the NPC essentially unhindered, at rates inversely related to their Stokes radius [8]: this passive NPC transit appears fast only for small molecules (e.g. ions, RNAs) and is already clearly delayed for GFP-sized proteins [9], [10]. Large molecules and molecular complexes (>60–70 kDa) transit the pore through active carrier-mediated, signal-dependent processes [11]. Whether a single NPC accommodates passive and active import processes by means of a common channel or separate channels is still a matter of great debate [12], [13].

A common way to address transport processes across the NE in living cells is based on the use of GFP-based bulk proteins. Untagged GFPs are commonly used as a benchmark for passive diffusion through the pore [10], [14], as they do not directly interact with the NPC components. The recognition motifs that are able to drive GFP molecules towards the nucleus are called Nuclear Localization Signals (NLS). NLS-bearing substrates bind in the cytoplasm to importins (namely Importin α and β, in the following: Impα and Impβ), soluble accessory proteins that mediate transient interactions with the FG proteic components of the pore [2], [15], [16]. Import complexes, consisting of transport substrates and importins, form in the cytoplasm and are dissociated after transit through the NPC by the nuclear RanGTP [17]. The asymmetric distribution of RanGTP between nucleus and cytoplasm provides the driving force leading to unidirectional active cargo transport [18]. According to their molecular size, NLS-bearing cargoes can concomitantly pass through the NPC by passive diffusion [10].

Current approaches to measure transport in cells based on perturbation methods like fluorescence recovery after photobleaching (FRAP) are invasive, fluctuation correlation methods (FCS) are local and SPT requires the observation of isolated particles for relatively long time. We recently introduced a method based on pair correlation functions [19] which measure the time the *same* molecule takes to migrate from one location to another within the cell. This approach builds on recent progress of using dual foci FCS [20] and combines FCS and SPT technologies providing single molecule sensitivity but in the presence of many molecules. Our method is based on spatio-temporal fluctuation correlation techniques in which several points in space are cross-correlated and the precise time required for the same molecule to be found in a given location away from the position at time zero can be measured. If there is a barrier to diffusion at any given location, it will require a longer time to find the particle at a position across the barrier, as demonstrated for the diffusion of molecules in cellular membranes [19].

Here we show the applicability of the pCF method to the 3D interior of the cell by addressing intracellular transport. To this end we used a prototypical protein substrate undergoing carrier-mediated transport (as described above), composed of a 27-kDa GFP moiety linked to the functional NLS of simian virus 40 (SV40) large tumor/antigen. By the pCF approach we are able to track the *same* NLS-GFP molecule in a sea of many other fluorescently labeled molecules, with high temporal resolution (in the millisecond scale) and spatial resolution (limited by diffraction). Our results highlight both the heterogeneity of NLS-GFP intracompartment diffusion and the presence of the nuclear envelope barrier to its transport between compartments. Concerning the latter process, we observed and characterized the kinetics of NLS-GFP bidirectional passive diffusion and cytoplasm-to-nucleus active import at the single molecule level, and in living, unperturbed cells.

## Results

### The NLS-GFP model substrate

**Figure 1:**
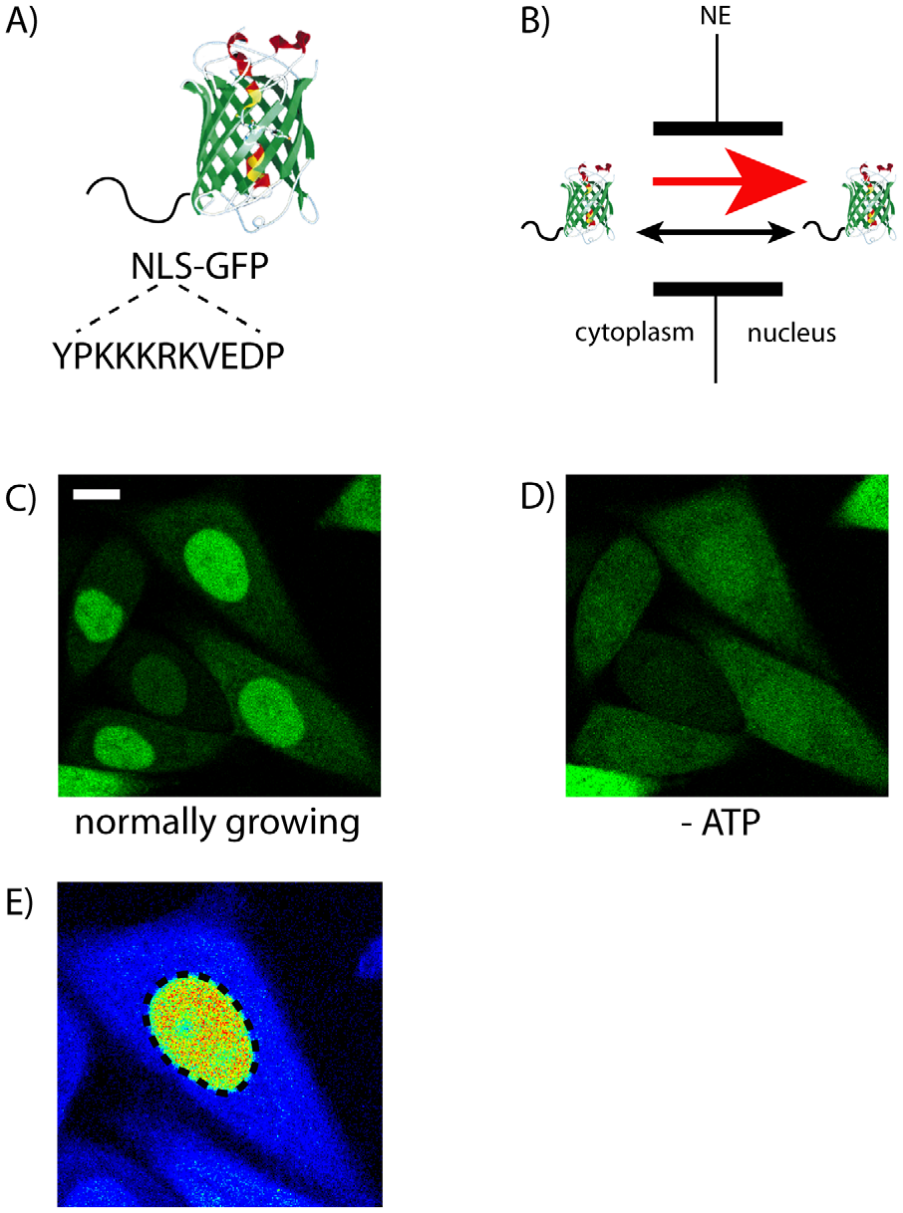
Overview on the model system used in this study. (A) The model substrate we studied is composed by a GFP fused to the functional NLS of SV40. (B) NLS-GFP is recognized by specific receptors in the cytoplasm and actively transported into the nucleus (red arrow). Concomitantly, it can passively diffuse across NPCs in both directions (black arrow), as its size (∼28 kDa) is below the cut-off limit of nuclear pores. (C) NLS-GFP intracellular localization upon transient transfection, and under normal growing conditions. Scale bar: 10 µm. (D) Upon energy depletion, NLS-GFP is homogeneously distributed across NE, as cytoplasm-to-nucleus active import is impaired. (E) Magnification of one cell shown in (C), with the NE highlighted by a dotted line.

We used a prototypical protein substrate for carrier mediated nuclear import, composed by a 27-kD-GFP moiety fused to the functional NLS of SV40 (sequence: YPKKKRKVEDP, Fig. 1A). This GFP cargo can actively cross the NE owing to the NLS-driven carrier-mediated processes and concomitantly shuttle between compartments by passive diffusion, as its molecular size is below the 60-70-kD-size exclusion limit of the NPC (Fig. 1B). The resulting physiological localization upon transient transfection into CHO-K1 cells is shown in Fig. 1C (K_eq_  =  nuclear concentration/cytoplasmic concentration = 6.5±1.7 in the observed cells). The observed behavior is consistent with the presence of cytoplasm-to-nucleus NLS-driven import: when this process is blocked by energy depletion, NLS-GFP passive diffusion to the cytoplasm can successfully restore a homogeneous protein distribution across NE (Fig. 1D). Images like that shown in Fig. 1C were used to determine the position of the NE (dotted line in Fig. 1E), on the basis of the NLS-GFP sub-cellular localization.

### The ACF-carpet analysis

**Figure 2:**
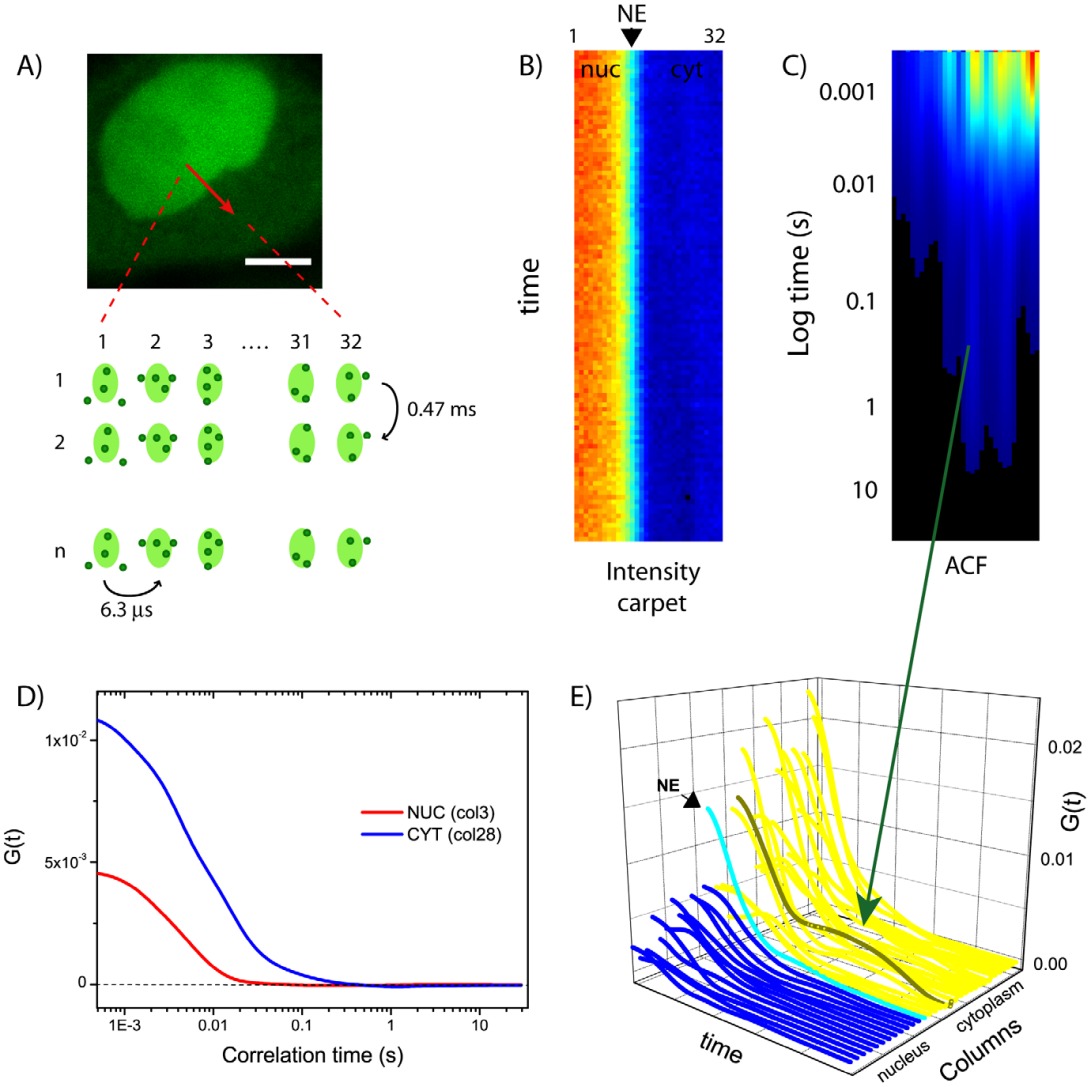
ACF carpet analysis. (A) A 3.2 µm-line across NE is scanned left-to-right with a pixel dwell time of 6.3 µs (32 pixels) and a line time of 0.47 ms. Scale bar of the image: 5 µm. (B) Fluorescence intensity is represented as a carpet with the line in the x-direction and the time in the y-direction. (C) The ACF is calculated pixel-by-pixel along the scanned line and represented as a carpet. (D) Two columns extracted from the ACF carpet are displayed, one corresponding to the nucleus (col. 3, in red), the other to the cytoplasm (col. 28, in blue). (E) 3-D-plot of the total ACF carpet.

As schematically represented in Fig. 2A, data are collected along a 3.2-µm-line across the NE, scanned from nucleus to cytoplasm with a sampling rate of 6.3 µs/pixel and 0.47 ms/line. Data are presented under the form of an intensity carpet in which the *x*-coordinate represents the position along the line (pixels) and the vertical coordinate corresponds to the time of acquisition (successive lines) (Fig. 2B). By sampling fluorescence at low laser power at the sample, we were able to collect up to about 2×10^5^ scan lines (corresponding to approximately 90 s) with no detectable photobleaching on the sample. Then we selected smaller regions (corresponding to ∼30 s) in order to minimize the average changes of fluorescence intensity over time in the sample (e.g. average changes in the position of the NE barrier, see Materials and Methods).

There is a direct relationship between the position of a point in the carpet-representation and the time the intensity is acquired at that point. Thus, we can calculate the autocorrelation function (ACF) for each pixel along the line (Fig. 2C). If we extract and fit a column of the ACF carpet, we can derive the NLS-GFP diffusion coefficient local to that pixel along the line, as it is conventionally done in a single-point FCS experiment. In Fig. 2D we report two example columns, corresponding to the cytoplasm (*blue*) and the nucleus (*red*). The maximum of amplitude of the ACF function (G(0)) is at time zero (since the particles correlate in the beam waist) and is inversely proportional to the number of molecules in the pixel: accordingly, the cytoplasmic column yields an higher G(0)-value (lower number of molecules) compared to the one extracted from the nucleus. In this particular case, the fitting procedure yields D = 11.6±1.5 µm^2^/s for the nuclear curve (*red*), and D = 3.4±0.6 µm^2^/s for the cytoplasmic one (*blue*): these values are representative of the characteristic NLS-GFP diffusion coefficients in the two compartments, that can be obtained by fitting the average of all the nuclear (or cytoplasmic) ACF columns in all the observed cells (Table 1). Furthermore, the reported average D-values were independently confirmed by raster image correlation spectroscopy (RICS) measurements performed on NLS-GFP in the same cell line (see Table S1).

**Table 1:** Diffusion coefficients derived by ACF-carpet fitting (µm^2^/s).

	Nucleus	Cytoplasm
**NLS-GFP**	12.5±4.5	5.5±3.2

Average diffusion coefficients (‘D’, µm^2^/s) calculated separately in the nucleus and the cytoplasm of N = 8 observed cells. For each cell, the ACF curves from all nuclear (and cytoplasmic) points were averaged and fitted to the 3D equations of diffusion. Single-cell D-values were then averaged to obtained the cumulative values displayed here (mean ± sd).

The 3-D-plot of the ACF carpet (Fig. 2E) highlights the heterogeneity of the intracellular environment: the G(0)-values and the slope of the ACF vary pixel-by-pixel along the scanned line in both compartments. Moreover, this analysis reveals cytoplasmic sub-regions with a detectable slow diffusive component (an example is arrowed in Fig. 2E, see discussion).

### The pCF analysis detects heterogeneous intracompartment diffusion

**Figure 3:**
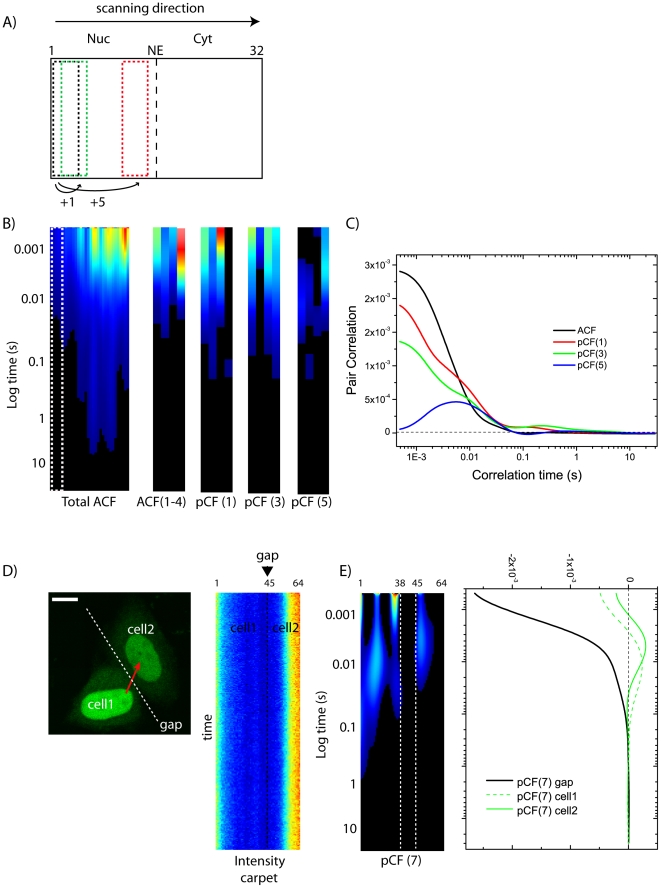
pCF analysis of intracompartment diffusion. (A) Schematic representation of intracompartment pCF analysis. (B) ACF and pCF(1, 3, 5) carpets corresponding to an intranuclear segment (columns 1–4). (C) For each carpet shown in (B), the average correlation curve is calculated and displayed in the graph. (D) Image of two neighbor cells with the scanned line (in this case 64 pixels = 6.4 µm) highlighted in red, and the corresponding intensity carpet along that line. Scale bar of the image: 5 µm. (E) pCF(7) analysis highlights the presence of the impenetrable gap between the two cells (approximately at position 45 along the line). The corresponding plot does not yield a maximum of correlation in the gap (even at very long times), while it shows the characteristic positive correlation within each cell. Note that the pCF analysis correctly yields an ‘apparent’ width of the gap that corresponds to the distance selected for the analysis (i.e. 7 pixels = 0.7 µm in Fig. 3D, gap from column 38 to 45 approximately).

The time cross-correlation between points along lines separated by a given distance determines the average time necessary for a molecule to traverse that distance. First we analyzed distances corresponding to NLS-GFP intracompartment diffusion. As schematized in Fig. 3A, a nuclear (or cytoplasmic) sub-region (in this case 3-pixel wide) is selected and cross-correlated to equivalent sub-regions at different distances along the scanned line, but within the same compartment. In Fig. 3B we report the pCF-carpet for those sub-regions, calculated at a distance of 0 (corresponding to the ACF), 1, 3, and 5 pixels along the line, by Eq. 3 (Materials and Methods). As shown in the average cross-correlation plot in Fig. 3C, when the distance is small points are within the PSF which produces correlation of the intensity fluctuations at very short time (e.g. pCF(1) curve). As the distance increases, the time cross-correlation curve starts at very low (even negative) amplitude and then increases with a maximum in amplitude which is due to the average transit time (diffusive or not) between the two locations (e.g. pCF(5) curve). The characteristics anti-correlation at short time and then the increase in the correlation at longer times is the signature that we are detecting the “same” molecule at a later time: this is the spatial equivalent of the “time anti-bunching” principle used to detect the fluorescence decay from the same molecule. The observed decrease of the cross-correlation function with distance is due to the angular spread of the particle trajectories (the amount of pair correlation decreases asymptotically as the square of the distance between the two points, for distances larger than the waist of the PSF). This kind of analysis has been performed in both the nuclear and cytoplasmic compartments, with similar results.

The average pair cross-correlation functions corresponding to intra-nuclear (or intra-cytoplasmic) NLS-GFP diffusion follow the general pattern of diffusing proteins, with expected average transit delays of few milliseconds (e.g. 1–30 millisecond range for pCF(4) calculated in the nucleus of N = 8 observed cells). However, several intra-compartment barriers to free isotropic diffusion can be detected along the line in pCF carpets: this is testified by the heterogeneity among columns in the same pCF carpet (see different columns in Fig. 3B). This result is not unexpected for the diffusion of proteins in the crowded and sub-compartmentalized intracellular environment (see discussion). These barriers are immobile during the time of acquisition (pCF was calculated averaging measurements for about 30 s), but molecules can easily go around the obstacle (they do not produce extensive lengthening of the time of the maximum).

As a control experiment for the observed delays, we imaged two cells in close proximity, adjacent one to another and possibly in contact: we placed the scan line across the supposed gap between cells, as shown in Fig. 3D. In this case NLS-GFP molecules are not allowed to cross the physical boundary of the cell membrane and the pCF algorithm should indicate lack of propagation of the molecules from one cell into the other. This is shown in Fig. 3E, where the pCF(7) carpet shows obvious boundaries along the columns corresponding to the gap region, while it detects the characteristic positive correlation in each cell. Not even at very long time we observe crossing over this impenetrable barrier (plot in Fig. 3E).

### Detecting obstacles to intercompartment diffusion: pCF analysis of nucleus-to-cytoplasm passive diffusion

**Figure 4:**
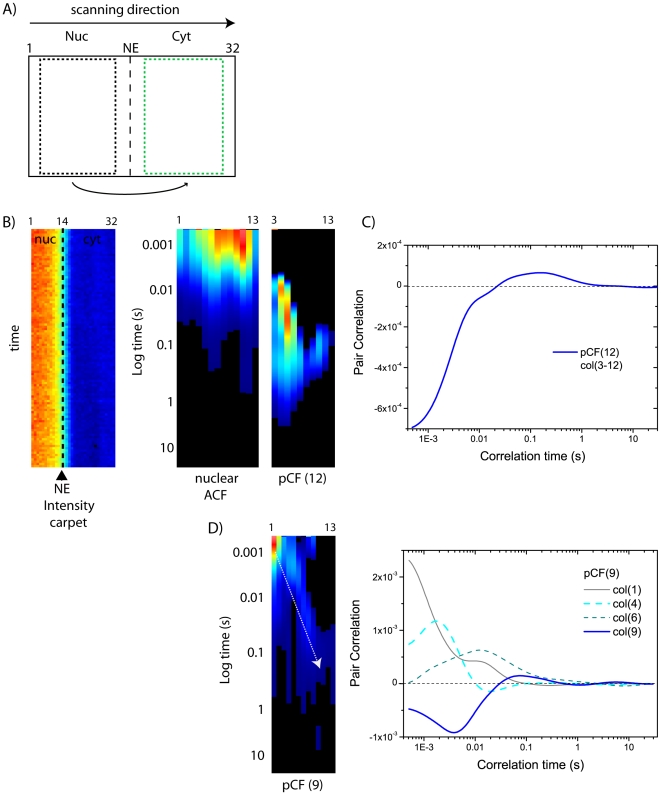
pCF analysis of nucleus-to-cytoplasm transport. (A) Schematic representation of intercompartment pCF analysis: scanning direction is from nucleus to cytoplasm (B) From the total intensity carpet (left panel) we select the nuclear compartment, calculate the ACF (middle panel), and the pCF function at a distance that entirely correlates with the cytoplasm (in this case pCF(12) for columns 3–12, right panel). (C) Average pCF(12) calculated for the carpet in (B). (D) pCF(9) calculated for the whole nuclear compartment. As shown in the graph, pCF(9) yields different average delays depending on the column chosen for analysis.

In the previous section we showed that the same molecule can be detected at a given distance from the original starting point, within each compartment. Here we address the nucleocytoplasmic transport by spatially cross-correlating regions across the NE barrier (scheme in Fig. 4A). In our measurements the laser scans in one direction (left-to-right) but we can choose the orientation of the pCF analysis and thus automatically select the process of interest across NE. First we selectively monitor nucleus-to-cytoplasm (no additional transport processes take place along this direction). To do this, we select the whole nuclear compartment (as all nuclear molecules will utilize the passive diffusion route to enter the cytoplasm) and cross-correlate it at a distance (in pixels) entirely corresponding to the cytoplasm (Fig. 4A). The pCF(12)-analysis reported in Fig. 4B and 4C (right panel) clearly shows the lengthening of the time of the maximum in correlation, compared to free intra-compartment diffusion. The average characteristic time for transporting NLS-GFP molecules across NE by passive diffusion falls between 100 and 500 ms (range of reported peak-values in N = 8 analyzed cells). We obtained similar results by measuring the transit delays of untagged GFP passively diffusing across NE in N = 5 analyzed cells (see Figure S1).

Overall, these results are fully consistent with the presence of the NE barrier to NLS-GFP nucleus-to-cytoplasm passive diffusion. The observed range of delays cannot be explained by assuming free diffusion between compartments, as it would produce much shorter transit times (few milliseconds, as shown above). This prediction is confirmed by the analysis reported in Fig. 4D: the pCF(9) carpet of the nuclear compartment shows delays of few milliseconds for pairs of pixels that cross-correlate within the nucleus (intracompartment diffusion), while it yields a clear lengthening of the mean NLS-GFP transit delay for pairs of pixels that cross-correlate across the NE (passive diffusion through the NPC). In this particular example, the pCF(9) analysis of column 9 (diffusion across NE) yields a maximum transit time that is approximately 50-folds larger compared to that obtained for column 4 (diffusion within the nucleus). In general, by this kind of analysis we found ratios ranging from 40 to 100.

### Detecting obstacles to intercompartment diffusion: pCF analysis of cytoplasm-to-nucleus active import

**Figure 5:**
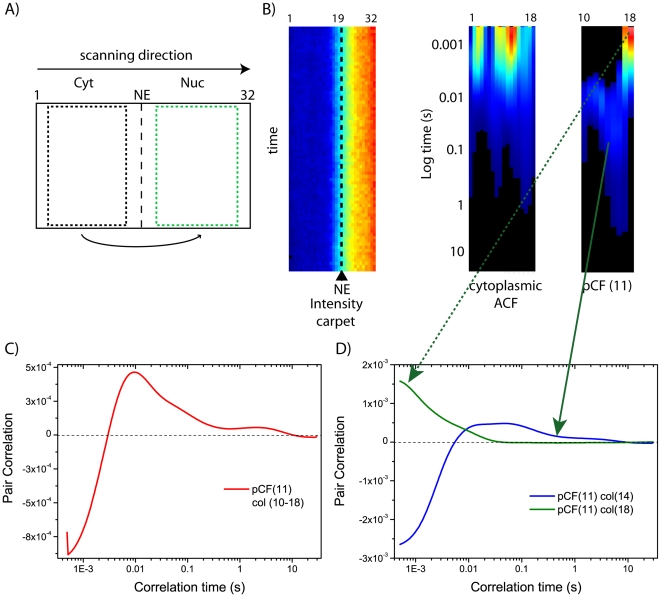
pCF analysis of cytoplasm-to-nucleus transport. (A) Schematic representation of intercompartment pCF analysis: scanning direction is from cytoplasm to nucleus. (B) From the total intensity carpet (left panel) we select the cytoplasmic compartment, calculate the ACF (middle panel), and the pCF function at a distance that entirely correlates with the nucleus (in this case pCF(11) for columns 10–18, right panel). (C) Average pCF(11) calculated for the carpet in (B). (D) pCF(11) of two columns extracted from carpet shown in (B).

If we analyze NLS-GFP transport events taking place towards the nucleus (Fig. 5A), we can concomitantly analyze both passive diffusion and active import of NLS-GFP across NE (as depicted in Fig. 1B). From the total intensity carpet we can select the cytoplasmic compartment, and cross-correlate it to a distance (in pixels) entirely corresponding to the nucleus (Fig. 5B). As reported in the example of Fig. 5B (right panel), the pCF-carpet is clearly enriched in shorter delays (few milliseconds) compared to the same analysis in the opposite direction (shown in Fig. 4): accordingly, the corresponding average curve displays a maximum of correlation at approximately 10 milliseconds (Fig. 5C). This result is in keeping with the presence of a fast transport process taking place from the cytoplasmtothe nucleus (e.g. carrier-mediated nuclear import): overall, in this direction we reported pCF peak-values in the 1–40 ms range (N = 8 observed cells). Accordingly, we measured very short cytoplasm-to-nucleus transit times (1–30 ms range in N = 5 cells) for the Impβ-EGFP import carrier transfected into living cells (example shown in Figure S2).

Note that a slow correlation component is often visible when pCF is calculated along the cytoplasm-to-nucleus direction (example shown in Fig. 5C). This contribution arises from pixels in which we clearly detect delays in the range typical of molecules passively diffusing across NE (see blue arrowed column in Fig. 5D). It is worth noting that approaching the NE the pCF starts being dominated by very short delays (in the range of 1–10 ms, green arrowed curve in Fig. 5D), regardless of the correlation distance. This result can be related to the intracellular localization of endogenous Impα/β carrier proteins (see Discussion).

**Figure 6:**
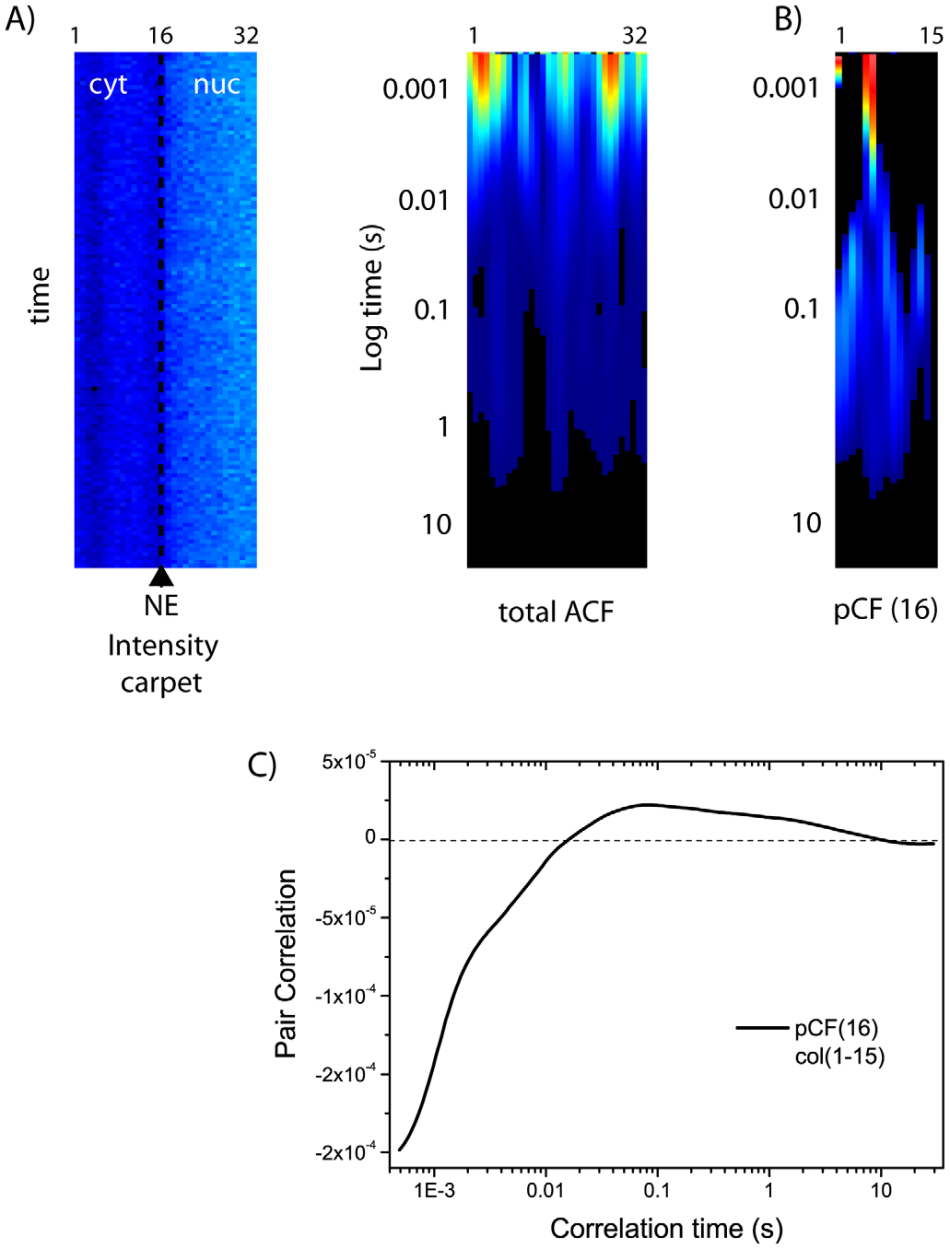
pCF analysis of cytoplasm-to-nucleus transport under energy-depleting conditions. (A) Intensity carpet and total ACF analysis for an ATP-depleted cell, with cytoplasm-to-nucleus scanning direction. (B) pCF(16) analysis of the cytoplasmic compartment. (C) Average correlation calculated from the carpet in (B).

As an additional control for the observed active-import delays, we performed measurements in energy depleted cells (Fig. 6). Under these conditions passive diffusion restores a homogenous NLS-GFP distribution across NE (intensity carpet in Fig. 6A), as it is the only route available for protein nucleocytoplasmic shuttling. Accordingly, the ACF analysis yields a quite homogeneous distribution of protein diffusivity across NE (Fig. 6B). Notably, by cross-correlating the two compartments (e.g. pCF(16) in Fig. 6C and D), we observe the typical transit delays of molecules undergoing passive diffusion through NPCs in both directions (150–1000 ms in N = 5 observed cells).

## Discussion

The pCF method is based on the spatio-temporal cross-correlation of the position of the same molecule at a given distance and at a given time. In our approach, we have many independent points along a line that can be correlated simultaneously to measure the anisotropic diffusion of molecules. This principle has the potential to track a large number of molecules and provide a true measurement of the diffusion of *single* proteins in cells [19].

Here we demonstrate for the first time the applicability of this method to the 3D interior of a living cell by addressing the prototypical nucleocytoplasmic transport of a transfected NLS-GFP protein (Fig. 1). Notably, the pCF analysis provides us with single-molecule sensitivity but in the presence of many molecules. This information cannot be obtained by ensemble averaging measurements such as FRAP or other image correlation spectroscopy methods, like RICS or STICS. Nuclear transport is a biological topic in which SPT measurements have given several quantitative results in both permeabilized [21], [22], [23] and living cells [24], with an unprecedented precision. These SPT measurements, however, rely on bright and isolated particles to be tracked many times in order to acquire enough statistics. Furthermore, most of the SPT experiments require the molecule of interest to be purified and labeled with large particles (gold, quantum dots) that can modify the overall transport dynamics of the protein. Instead, the pCF approach directly measures protein diffusion at the single-molecule level in living and unperturbed cells.

First, we calculated the autocorrelation function for each pixel of the scanned line (Fig. 2): by fitting the ACF we obtained the local NLS-GFP diffusion coefficient. The average nuclear and cytoplasmic NLS-GFP diffusion coefficients (12.5 and 5.5 µm^2^/s, respectively) were independently confirmed by RICS analysis (Table S1) and are in keeping with reported estimates based on FRAP [25]. Overall, the observed difference in the mean D-values between compartments (approximately a factor of two) is consistent with the reported pattern of NLS-specific interactions with the import machinery (NLS-Impα/β complex formation in the cytoplasm, complex dissociation in the nucleus) [26], [27]. The reported local variations of D-values along the scanned line also highlight the heterogeneity of the intracellular NLS-GFP diffusivity. This heterogeneity is not surprising, as both the nucleus and the cytoplasm are crowded environments, filled with several potential local restrictions to NLS-GFP diffusion (e.g. highly packed DNA, endoplasmic reticulum, organelles). Heterogeneity of diffusion is also observed in the ACF-profile of a single pixel, with some cytoplasmic sub-regions showing an additional slow diffusive component (example in Fig. 2E). This long-lasting correlation may originate from either a fraction of slowly diffusing NLS-GFP molecules or from average changes of fluorescence intensity in that particular region during the time of acquisition (e.g. movements of cytoplasmic organelles, photobleaching, etc.).

Next we applied the pair correlation analysis to the study of NLS-GFP intracompartment diffusivity (Fig. 3). As previously demonstrated [19], we reported the predicted anti-correlation at short time for spatial separations larger than the beam waist, the expected decrease of the overall amount of correlation as a function of distance and the existence of a maximum of correlation at a distance corresponding to the average time necessary to reach that location. We selected regions of the cell free of obvious features, but we found a relatively heterogeneous diffusion of NLS-GFP molecules (see differences among columns of the pCF-carpets in Fig. 3B), probably owing to the presence of several intracompartment barriers at the sub-microscopic level. On the average, these barriers do not produce extensive delays to intracompartment NLS-GFP movement (reported transit times are in the 1–30 ms range). This outcome also demonstrates the exquisite sensitivity of the pCF approach to the presence of obstacles to diffusion, an information not available with other classical non-imaging methods.

Next we addressed NLS-GFP nucleus-to-cytoplasm passive diffusion. The pCF algorithm nicely detects a considerable lengthening of the time of the maximum when two positions across NE are correlated (maximum of correlation in the 100–500 ms range). By comparing the same pCF-length calculated either across NE and within the same compartment, we can conclude that, on the average, NLS-GFP molecules are slowed down ∼40–100 folds when they pass through the pore (with respect to free intracompartment diffusion), in good agreement with previously reported estimates based on FRAP [14].

If we apply the pCF analysis along the cytoplasm-to-nucleus direction we can measure the contribution of active nuclear import to NLS-GFP intercompartment movement. In fact, although passive diffusion delays are still discernible, the pCF-carpet is clearly dominated by shorter transit times, characteristic of fast carrier-mediated transport. On the average, reported delays are in the 1–40 ms range, regardless of the analyzed pCF length: this outcome nicely matches with active translocation times measured by SPT techniques [21], [22], [23], [24]. It is worth noting that, in each experiment, transit times are spatially distributed according to the distance from the NE barrier. In particular, the pCF analysis yields the fastest transit times (and the largest number of transit events as well) near the NE barrier: this result is not surprising, as it correlates with the intracellular localization of Impα/β carriers, that are locally accumulated on the NE [27], [28]. We unequivocally linked this fast component to carrier-mediated active import by showing two internal controls: 1) the fast cytoplasm-to-nucleus component (1–30 ms) disappears in energy depleted cells, replaced by the characteristic transit delays of passively diffusing molecules; 2) the classical import carrier Impβ shows cytoplasm-to-nucleus transit times in the 1–30 ms range, in perfect agreement with the values reported for the NLS-GFP cargo.

Generally, we observed significant variations in the position of the peak of correlation among different experiments (particularly in those across NE) and a broad distribution of transit times around the maximum of correlation in each experiment. The first outcome can be explained by considering that we do not know the distance between the scanned line and the nearest pore channel of the nuclear envelope: the time position of the peak of correlation is expected to be strictly dependent on this parameter. Concerning the latter issue, it should be kept in mind that each time we correlate two points (for instance: A and B) along a line, we average the contribution of all the single trajectories (and transit times) allowed for molecules to go from A to B. Based on the pair correlation approach described here, we are currently envisioning a way to achieve ‘single-pore’ resolution to get new insight into the NPC crossing mechanism in living cells.

## Materials and Methods

### Cell culture, plasmids and treatments

CHO-K1 cells were grown in Ham's F12K medium supplemented with 10% of fetal Bovine Serum at 37°C and in 5% CO_2_. CHO-K1 cells were plated on imaging dishes and transiently transfected using Lipofectamine 2000 according to manufacturer's protocol. The plasmid encoding for GFP and NLS-GFP used here have been described in detail elsewhere [10]. Importinβ-GFP is a generous gift of Marilena Ciciarello (Istituto di Biologia e Patologia Molecolari, CNR, Roma). Details about the plasmid can be found in Ref. [28]. All the cells were imaged at room temperature. Energy depletion experiments were conducted by using sodium azide and 2-deoxy-d-glucose, as described elsewhere [10]. For the imaging of nuclei, cells were incubated with the DNA-staining Hoechst dye (5 µg/ml for 30 minutes) and then examined by confocal microscopy.

### Microscope

Microscopy measurements were performed with a Zeiss LSM 710 META laser scanning microscope. Data were acquired and processed by the SimFCS software developed at the Laboratory for Fluorescence Dynamics (www.lfd.uci.edu). A 63X water immersion objective (Zeiss, Germany) with 1.2 N.A. was used for the measurements. The volume of the point spread function (PSF) was calibrated by measuring the autocorrelation curve for 20 nM fluorescein in 0.01 M NaOH, which was fit in turn with a diffusion coefficient of 300 µm^2^/s. Typical values of *w_0_* (that define the PSF) were in the range of 0.2 µm to 0.3 µm, depending on the laser wavelength and objective used. The average power at the sample was maintained at the milliwatt level. The following collection ranges were adopted: 400–450 nm (Hoechst), 500–540 nm (GFP).

### Description of the experimental setup

In our experimental setup we acquire data by rapidly scanning a diffraction limited laser beam (488 nm) along a line focused in the interior of the cell. The fluorescence intensity is sampled at a rate such that spatial locations along the line are oversampled with respect to the waist of the laser beam. For example, for a typical waist of the diffraction limited spot of 200 nm. As this spot moves along the selected line, we sample the intensity approximately every 100 nm (pixel size). In our set up we can scan along a line at the maximum speed in about 0.473 ms (∼2120 lines/s): in detail, the intensity is sampled every 6.3 µs on a 32 (or 64)-points line. As the distance between successive points is 100 nm, we can collect points along a line that is about 3.2 (or 64) µm long. This length scale is adequate for studying molecular transport across the NE, from about 200 nm to microns and in a time scale from microseconds to several minutes. 2×10^5^ consecutive lines (with no intervals between lines) are usually acquired. Then we select regions (∼6.4×10^4^ lines, corresponding to ∼30 s) with no average changes of fluorescence intensity (e.g. average changes in the position of the NE barrier over time and/or photobleaching). Such events are easily identifiable, as they give a pCF-profile without a minimum at early times since the correlation extends over many pixels.

### Data analysis and presentation

Data acquisition and calculation of the pair-correlation functions were done using the SimFCS software (Laboratory for Fluorescence Dynamics). Documentation for the SimFCS software can be found at www.lfd.uci.edu. Intensity data are presented using a carpet representation in which the *x*-coordinate corresponds to the point along the line (pixels) and the *y*-coordinate correspond to the time. The autocorrelation function (ACF) and the pair correlation functions at a given distance in pixels (pCF(pixel)) are displayed in pseudocolors in a image in which the *x*-coordinate correspond to the point along the line and the vertical coordinate correspond to the autocorrelation time in a *log*-scale. We analyzed 8 cells expressing NLS-GFP in physiological conditions, 5 cells expressing NLS-GFP in energy depleting conditions, 5 cells expressing untagged GFP and 5 expressing Impβ-GFP (both in physiological conditions). For each cell we measured intra-compartment and inter-compartment diffusion along a line across NE. For each calculated pCF(pixel = distance) we selected the time-value corresponding to the maximum of correlation (peak of the pair correlation curve). Cumulative transit delays are described in the text by the range of the obtained peak time-values in the whole population of cells (from the minimum to the maximum value obtained). In the figures we show single cell measurements that are representative of the cumulative results.

### Derivation of the pCF for diffusing particles

The diffusion propagator is given by Eq.1:
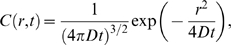
(1)where *C*(*r*,*t*) can be interpreted as being proportional to the probability of finding a particle at position *r* and time *t* if the particle is at position 0 at time *t* = 0. The fluorescence intensity at any given time and position *δr* from the origin is given by:
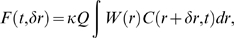
(2)where it is assumed that the fluorescence is proportional to the concentration, quantum yield *Q*, excitation-emission laser power, filter combination, and the position of the particle in the profile of illumination described by *W*(*r*). The pCF for two points at a distance *δr* as a function of the delay time *τ* is calculated using the expression:
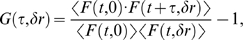
(3)


As in normal FCS, the pCF can be calculated analytically only for special cases of the profile of illumination function. In our case it was assumed that the illumination profile was described by a symmetric 2D Gaussian function. Estimation of the errors can be done using the data of our original manuscript (ref 19) where we measured the diffusion of beads in solution. Under these conditions, the diffusion is isotropic so that each point along the line should give the same result since the delay is uniform. Each curve has a hump corresponding to the average delay of the beads to reach a given distance. The standard deviation of each point of the pCF function is on the order of 5% of the size of the hump (see Figure 4 of reference 19). However, the average position of the hump can be determined with better precision. We use only the average position (in time) of the hump for our analysis.

## Supporting Information

Figure S1 (A) Schematic representation of a GFP molecule passively diffusing across the NPC. (B) Intracellular localization of transfected GFP in living CHO-K1 cells (left panel). To localize the position of the nuclear envelope we stained the nuclear compartment using the Hoechst dye (right panel), as described in the Methods section. The scanned line is depicted in red. Scale bar: 10 µm. (C) From the total intensity carpet (left panel) we selected the cytoplasmic compartment and calculated the pCF function at a distance that entirely correlates with the nucleus (in this case pCF(16) for columns 1–16, right panel). (D) Average pCF(16) calculated for the carpet shown in (C) (solid black line). The dashed line refers to the same pCF analysis conducted backwards, from nucleus to cytoplasm. As expected, in both directions we observe transit delays typical of passive diffusion.
(1.10 MB TIF)

Figure S2 (A) Schematic representation of a Impβ-GFP molecule crossing the NPC. (B) Intracellular localization of transfected Impβ-GFP in living CHO-K1 cells (left panel). The position of the NE is clearly discernible, due to the typical accumulation of Impβ-GFP on its NPC binding sites. The scanned line is depicted in red. Scale bar: 10 µm. (C) From the total intensity carpet (left panel) we selected the cytoplasmic compartment and calculated the pCF function at a distance that entirely correlates with the nucleus (in this case pCF(17) for columns 1–15, right panel). (D) Average pCF(17) calculated for the carpet shown in (C) (solid red line). As expected for a protein involved in the nuclear import process, we observe fast transit delays, typical of active translocation across NPC.
(1.24 MB TIF)

Table S1 Average diffusion coefficients (‘D’, µm2/s) calculated separately in the nucleus and the cytoplasm of N = 10 observed cells by Raster Image Correlation Spectroscopy. For each cell, the autocorrelation function for the nucleus (and cytoplasm) was fitted to the 3D equations of diffusion. Single-cell D-values were then averaged to obtained the cumulative values displayed here (mean ± sd).
(0.03 MB DOC)
