# Supplementary figures and images for: In Vivo Imaging of Single-Molecule Translocation Through Nuclear Pore Complexes by Pair Correlation Functions
Source: PLoS One. 2010 May 3;5(5):e10475. doi: 10.1371/journal.pone.0010475 (PMC2862743; doi:10.1371/journal.pone.0010475)

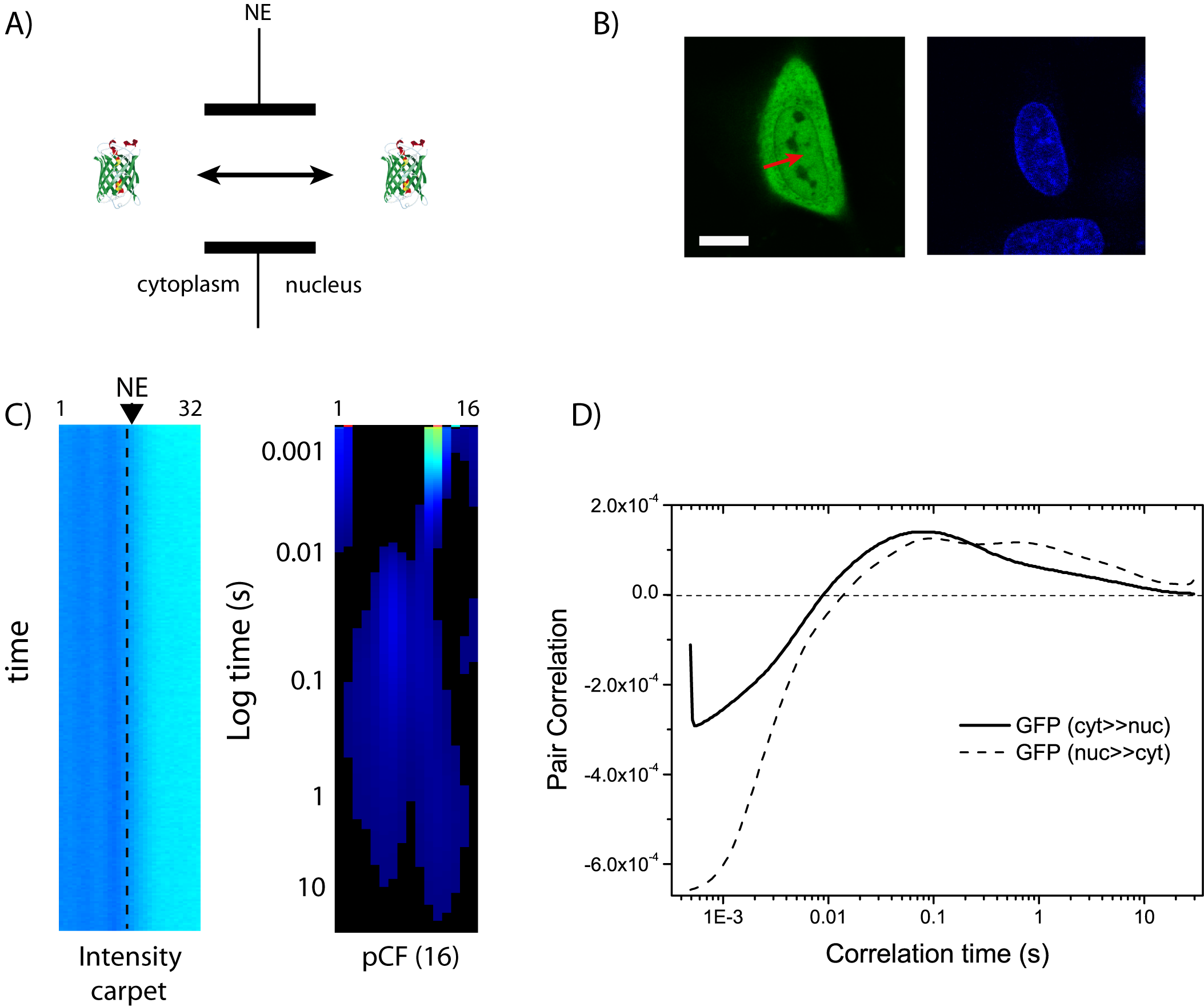

Supplement: Figure S1 — (A) Schematic representation of a GFP molecule passively diffusing across the NPC. (B) Intracellular localization of transfected GFP in living CHO-K1 cells (left panel). To localize the position of the nuclear envelope we stained the nuclear compartment using the Hoechst dye (right panel), as described in the Methods section. The scanned line is depicted in red. Scale bar: 10 µm. (C) From the total intensity carpet (left panel) we selected the cytoplasmic compartment and calculated the pCF function at a distance that entirely correlates with the nucleus (in this case pCF(16) for columns 1–16, right panel). (D) Average pCF(16) calculated for the carpet shown in (C) (solid black line). The dashed line refers to the same pCF analysis conducted backwards, from nucleus to cytoplasm. As expected, in both directions we observe transit delays typical of passive diffusion. (1.10 MB TIF) [file pone.0010475.s001.tif]

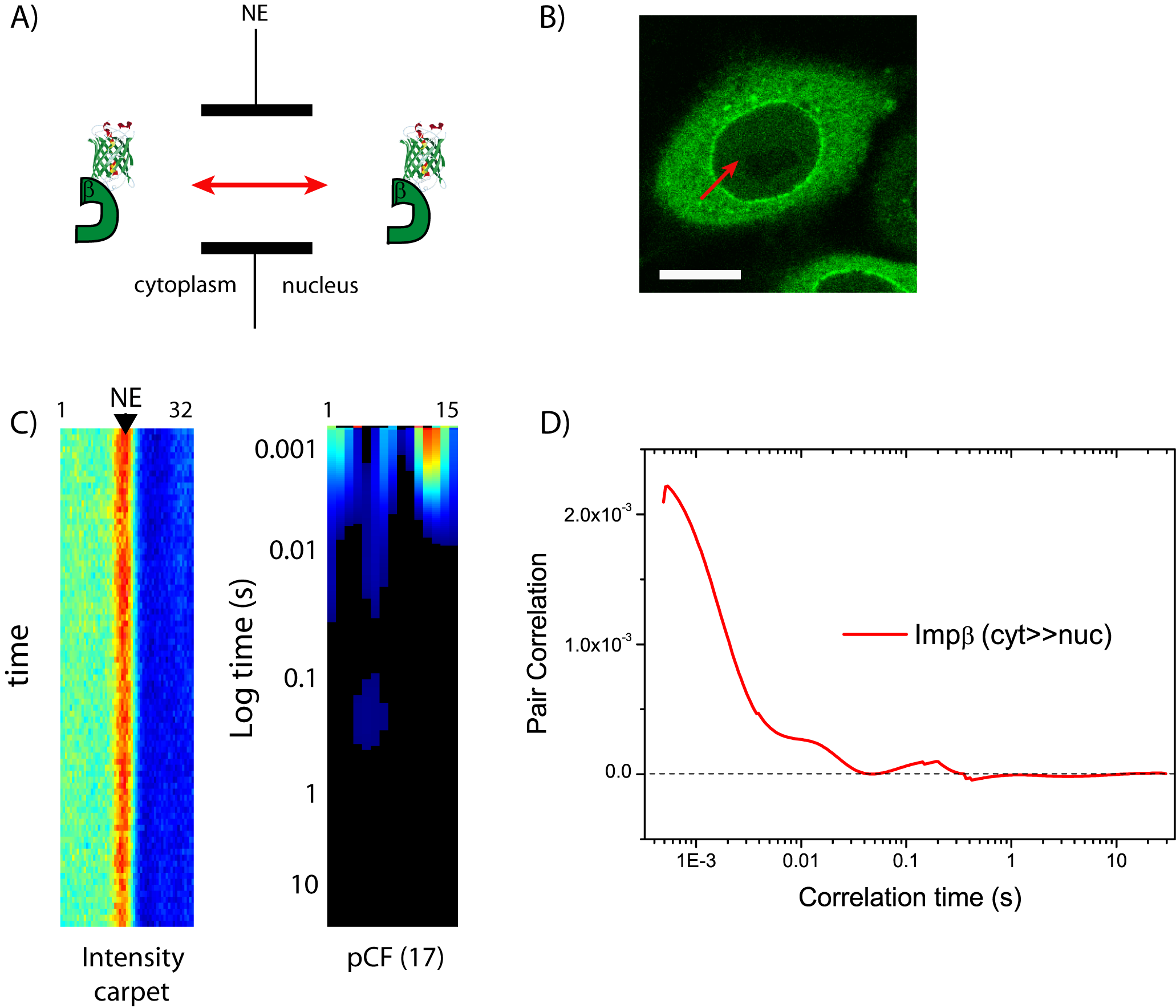

Supplement: Figure S2 — (A) Schematic representation of a Impβ-GFP molecule crossing the NPC. (B) Intracellular localization of transfected Impβ-GFP in living CHO-K1 cells (left panel). The position of the NE is clearly discernible, due to the typical accumulation of Impβ-GFP on its NPC binding sites. The scanned line is depicted in red. Scale bar: 10 µm. (C) From the total intensity carpet (left panel) we selected the cytoplasmic compartment and calculated the pCF function at a distance that entirely correlates with the nucleus (in this case pCF(17) for columns 1–15, right panel). (D) Average pCF(17) calculated for the carpet shown in (C) (solid red line). As expected for a protein involved in the nuclear import process, we observe fast transit delays, typical of active translocation across NPC. (1.24 MB TIF) [file pone.0010475.s002.tif]
