# Supplementary material for: In Vivo Imaging of Single-Molecule Translocation Through Nuclear Pore Complexes by Pair Correlation Functions
Source: PLoS One. 2010 May 3;5(5):e10475. doi: 10.1371/journal.pone.0010475 (PMC2862743; doi:10.1371/journal.pone.0010475)
Supplement: Table S1 — Average diffusion coefficients (‘D’, µm2/s) calculated separately in the nucleus and the cytoplasm of N = 10 observed cells by Raster Image Correlation Spectroscopy. For each cell, the autocorrelation function for the nucleus (and cytoplasm) was fitted to the 3D equations of diffusion. Single-cell D-values were then averaged to obtained the cumulative values displayed here (mean ± sd). (0.03 MB DOC) [file pone.0010475.s003.doc]

**Diffusion coefficients derived by RICS (μm^2^/s)**

|  | **Nucleus** | **Cytoplasm** |
| --- | --- | --- |
| **NLS-GFP** | 11 ± 5 | 4.5 ± 3 |
